# Median sternotomy approach for the repair of esophageal atresia: a case report

**DOI:** 10.1186/s40792-022-01523-5

**Published:** 2022-09-08

**Authors:** Hiroaki Fukuzawa, Mitsumasa Okamoto, Yudai Tsuruno, Ayako Maruo

**Affiliations:** 1 Department of Pediatric Surgery, Japanese Red Cross Society Himeji Hospital, 1-12-1 Shimoteno, Himeji, Japan; 2 Department of Cardiovascular Surgery, Kakogawa City Hospital, Kakogawa, Japan

**Keywords:** Esophageal atresia, Mediastinum, Median sternotomy, Tracheoesophageal fistula

## Abstract

**Background:**

Repair of esophageal atresia is usually performed through the right thoracic cavity. However, when the upper pouch of the esophagus and tracheoesophageal fistula (TEF) is located in the thoracic inlet and completely on the left side of trachea, it is difficult to dissect and anastomose the esophagus through the right thoracic cavity. We present a case of esophageal atresia, with the esophageal upper pouch located high and completely on the left side of trachea, successfully repaired via the median sternotomy approach.

**Case presentation:**

A male neonate with a birth weight of 1766 g was prematurely delivered via cesarean section at 34 weeks of gestation. Contrast-enhanced computed tomography (CT) showed that the upper pouch of the esophagus was located at the thoracic inlet and completely on the left side of the trachea; hence, a diagnosis of esophageal atresia was made. Moreover, a TEF was connected to the trachea at the level of the lower end of the upper esophageal pouch. An aberrant right subclavian artery and persistent left superior vena cava were also detected. Esophageal dissection and anastomosis were determined to be very difficult if approached from the right thoracic cavity. Therefore, we performed median sternotomy one day after the neonate was born. The upper pouch of the esophagus and TEF were easily dissected via the median sternotomy approach. Anastomosis of the esophagus was performed, with a good visual field, to the left of the trachea. The postoperative course was uneventful.

**Conclusions:**

This is the first reported case of a median sternotomy approach for esophageal atresia. This technique may be useful when a right thoracic approach is difficult, especially if the esophageal upper pouch is located completely to the left of the trachea or if it is higher than the normal position.

## Background

Repair of esophageal atresia (Gross C) is usually performed through right-sided thoracotomy or thoracoscopy [1]. However, left-sided thoracotomy can be indicated in the case of a right aortic arch. When the upper pouch of the esophagus is located at a high level, surgical manipulation is difficult with a thoracic approach on either the right or left side. Kemmotsu et al. reported a cervical approach in the case of an esophageal upper pouch with a tracheoesophageal fistula (TEF) located at an extremely high cervical position [2]. However, if both the upper pouch of the esophagus and the TEF are located around the thoracic inlet, it is difficult to dissect and anastomose them via the thoracic cavity or through a cervical incision. In this report, we describe a case of esophageal atresia, with the upper pouch of the esophagus and the TEF located in the thoracic inlet completely on the left side of the trachea, which was repaired via the median sternotomy approach. To our knowledge, this is the first reported case of a median sternotomy performed for the repair of esophageal atresia.

## Case presentation

**Fig. 1:**
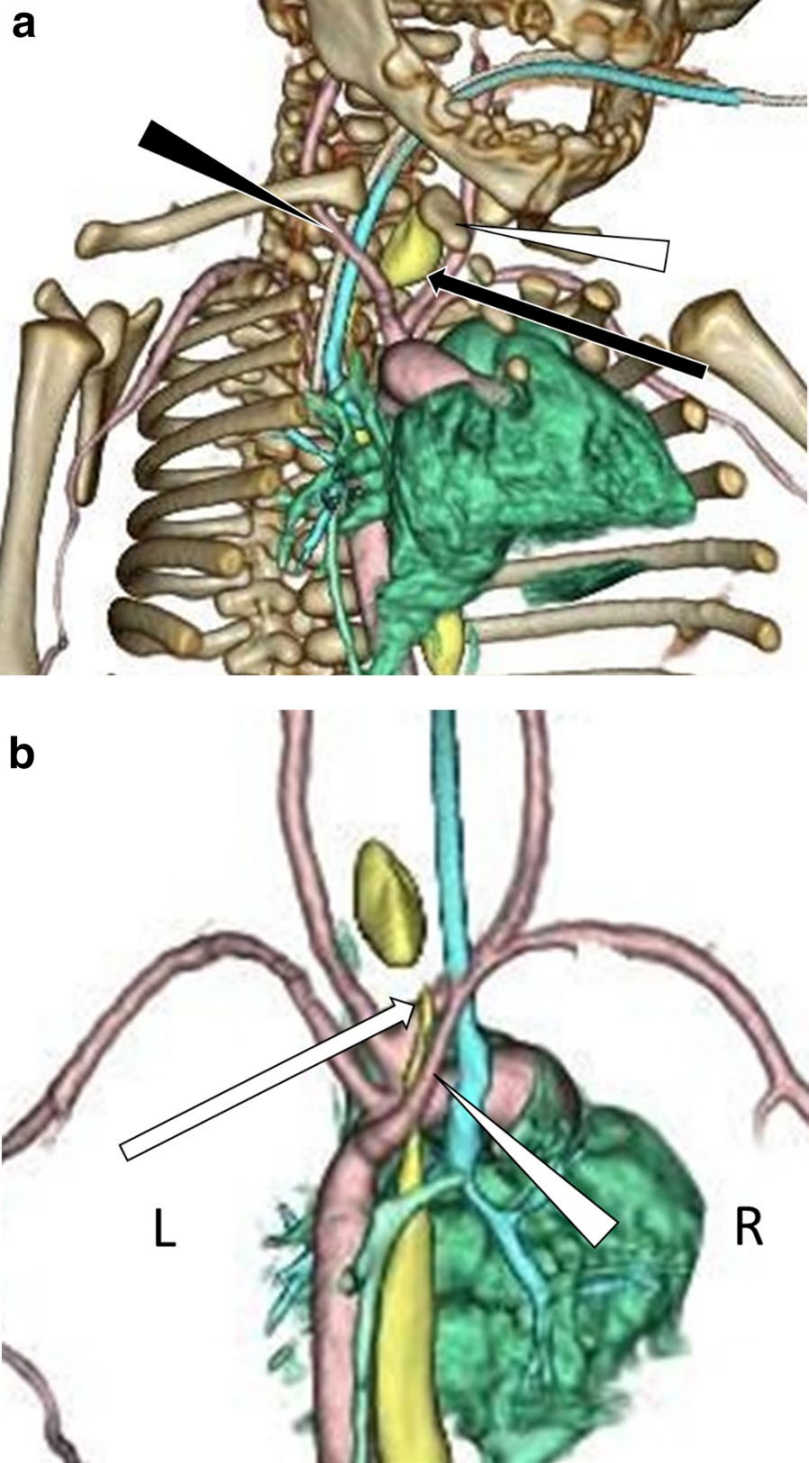
Three-dimensional computed tomography. The trachea (light blue), esophagus (yellow), and blood vessels (pink) are shown. **a** Image from the front right. The upper pouch of the esophagus (black arrow) was located at the thoracic inlet and completely to the left of the trachea. The brachiocephalic artery is indicated by the black arrowhead and the manubrium of the sternum is indicated by the white arrowhead. **b** Image from the back. The TEF indicated by a white arrow, was connected to the trachea at the level of the lower end of the upper pouch of the esophagus. The aberrant right subclavian artery is indicated by a white arrowhead

A male neonate with a birth weight of 1766 g was delivered prematurely via cesarean section at 34 weeks of gestation because of fetal distress. The patient was intubated and placed on a ventilator owing to poor respiratory status. Attempts at passing a nasogastric tube failed, and a diagnosis of esophageal atresia (Gross type C) was made. Contrast-enhanced computed tomography (CT) was performed to confirm the location of the upper pouch of the esophagus, TEF, and blood vessels. CT revealed that the upper pouch of the esophagus was located at the thoracic inlet (at the level of the first thoracic vertebra), completely to the left of the trachea (Fig. 1a). The TEF was connected to the trachea at the level of the lower end of the upper esophageal pouch (Fig. 1b). Furthermore, an aberrant right subclavian artery and persistent left superior vena cava were detected (Fig. 1b).

A right-sided thoracotomy is the usual surgical approach in such cases; however, the upper pouch of the esophagus would have been too high and far left for dissection and anastomosis. Moreover, the right aberrant subclavian artery appeared to interfere with the manipulation of the esophagus. If approached from the left thoracic cavity, the left internal carotid artery, subclavian artery, and aortic arch would interfere with the operative field. If a cervical incision was made, the upper esophageal pouch and TEF would be hidden behind the sternum and difficult to visualize and manipulate. Therefore, a median sternotomy approach was selected.

**Fig. 2:**
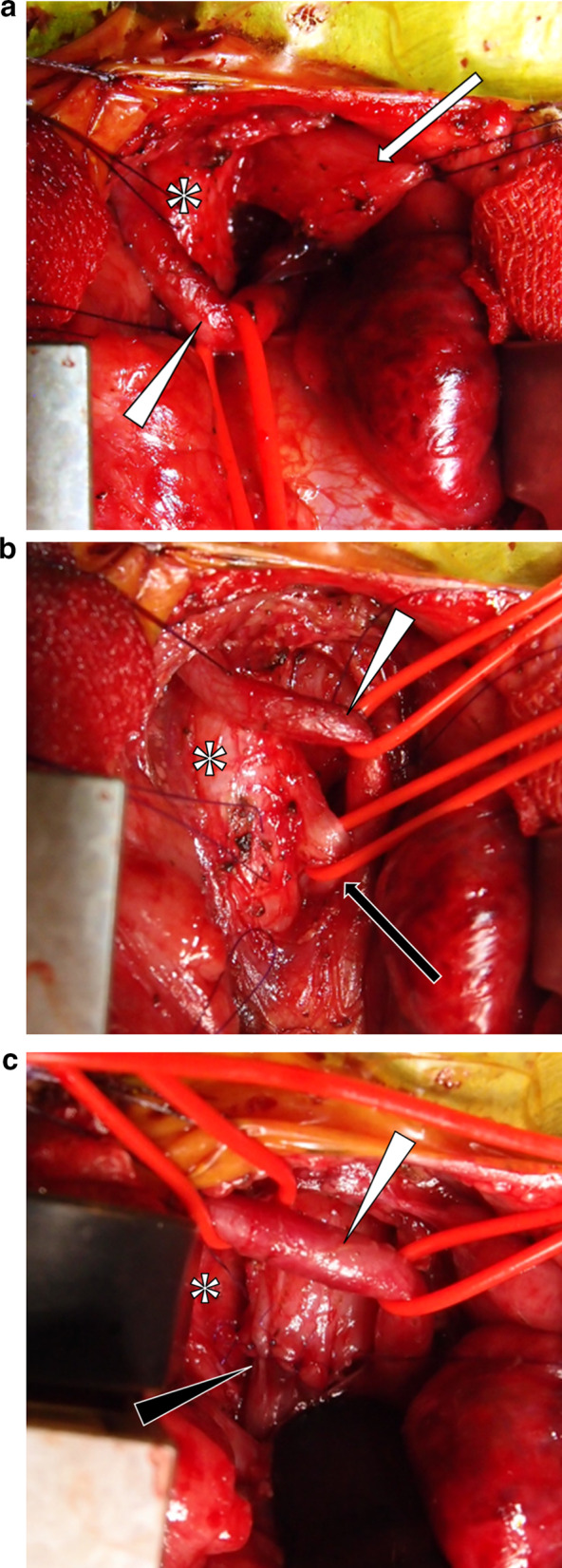
**a**–**c** Photographs of the surgical field. The upper pouch of the esophagus (white arrow), the trachea (white asterisk), the brachiocephalic artery (white arrowhead), the TEF (black arrow), and anastomosis of the esophagus (black arrowhead)

**Fig. 3:**
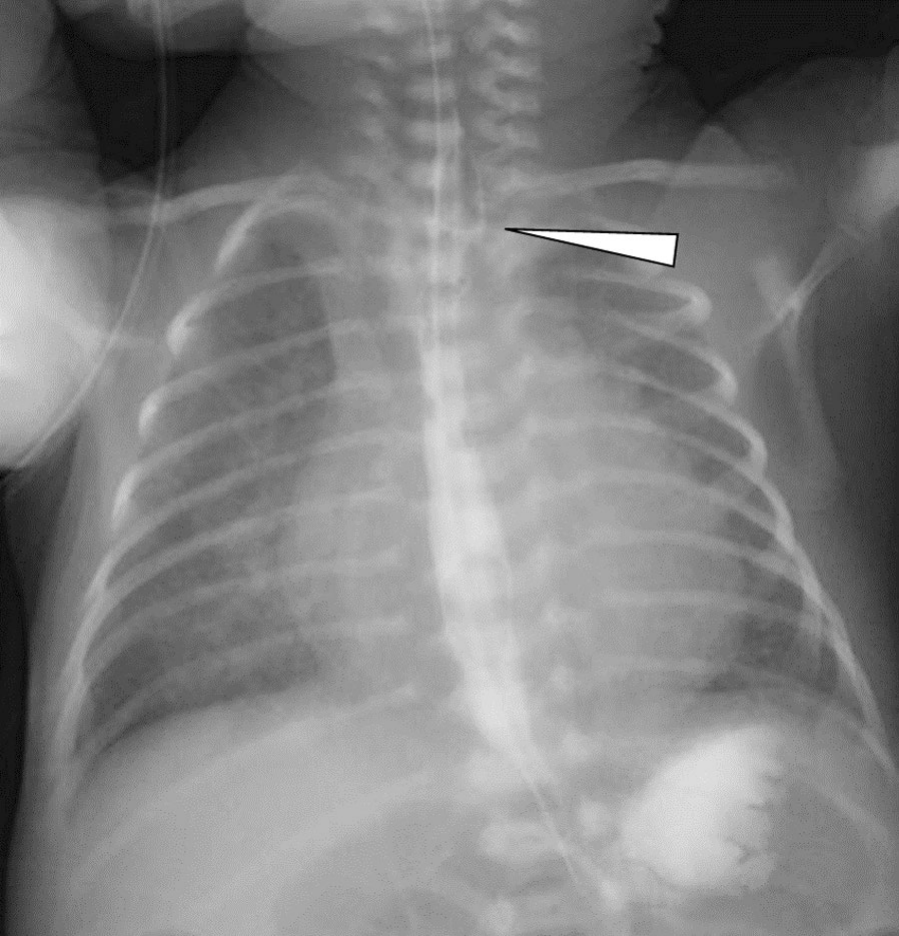
Postoperative esophagogram, showing anastomosis of the esophagus (white arrowhead). There was no noted stenosis at the anastomosis site

The operation was performed on the day after the neonate was born. The patient was placed in the supine position and a skin incision was made at the midline from the sternal notch to the xiphoid process. A median sternotomy was performed as for a cardiac operation. The thymus was divided at the midline, and the brachiocephalic artery, located in front of the trachea was dissected and taped. Fortunately, the left brachiocephalic vein that would have obstructed the operative field was absent owing to a persistent left superior vena cava. The upper pouch of the esophagus was observed to the left of the trachea and easily dissected (Fig. 2a). By placing a traction suture at the left side of the trachea and rotating the trachea to the right, the TEF connected to the dorsal aspect of the trachea was easily visualized and the lower esophagus was taped (Fig. 2b). The TEF was divided and the upper and lower sections were anastomosed with a good operative field on the left side of the trachea (Fig. 2c). A 6-Fr nasogastric tube was passed through the anastomosis to the stomach. A drainage tube was placed behind the sternum, which was sutured with absorbable sutures. The recurrent nerve was not identified at the time of operation, but no postoperative vocal cord paralysis was noted. The postoperative course was unremarkable, and no stenosis was noted (Fig. 3).

## Discussion

During the operation for esophageal atresia, when the upper pouch of the esophagus is located to the left of the trachea or in a very high position, identification, and dissection of the upper esophagus through the right thoracic cavity may be difficult. Furthermore, when the right aberrant subclavian artery is involved, as in our case, it obstructs the visual field, complicating surgical manipulation. Owing to these conditions in the current case, we performed the operation through a median sternotomy, which allowed easy manipulation with an unobstructed operative visual field. Normally, the left brachiocephalic vein is located in front of the trachea during a median sternotomy. However, in this case, owing to a persistent left superior vena cava, only the brachiocephalic artery was present in that position, which simplified the approach.

In previous cases, a cervical incision was used when the upper esophagus and TEF were located at a higher position than normal [2]. However, in cases where the upper esophagus and TEF were located near the thoracic inlet and dorsal to the sternum, a cervical incision is likely to provide a poor operative field, and a median sternotomy, as in the present case, is a better alternative. The median sternotomy approach is a far better option than the cervical approach in terms of the visual field. As this was the first time this approach was used for esophageal atresia, we performed a total sternotomy; however, a partial sternotomy may also be adequate.

Recently, CT has been proposed for newborns with esophageal atresia to identify the position of the TEF and anomalies of the aortic arch [3]. However, experience with the use of these diagnostic tools in the preoperative evaluation of neonates with esophageal atresia is very limited, and concerns have been raised about neonatal transport to the radiology department, the need for sedation, and CT-related radiation injury [4].

We routinely perform preoperative contrast-enhanced CT when the patient's condition permits. In the present case, the preoperative CT images allowed us to visualize the route to the upper esophagus and TEF, as well as the anastomotic maneuver. Contrast-enhanced CT provides detailed information on the location of the upper esophagus and TEF, as well as anomalies in the vasculature, which can be useful in performing surgery.

The median sternotomy approach may be indicated in particular cases, such as when the upper esophagus is extremely high (at the level of the thoracic inlet) and located to the left of the trachea. Moreover, the presence of a right aberrant subclavian artery complicated the approach from the right thoracic cavity. The disadvantages of a median sternotomy approach for esophageal atresia compared to a thoracic approach are cosmetic inferiority and the possibility of severe mediastinitis due to anastomosis leakage.

## Conclusions

In this report, we described a case of esophageal atresia that was repaired through a median sternotomy approach. This approach may be indicated when a right thoracic approach is difficult because of the high position of the upper pouch of the esophagus or TEF, or if these structures are located completely to the left of the trachea.

## Data Availability

The data are not available for public access because of patient privacy concerns, but are available from the corresponding author on reasonable request.

## Abbreviations

TEF: Tracheoesophageal fistula
CT: Computed tomography

## References

[1] Lewis S, Ashcraft KW, Holcomb GW, Murphy JP (2005). Esophageal atresia and Tracheoesophageal Malformations. pediatric surgery.

[2] Kemmotsu H, Joe K, Nakamura H, Yamashita M (1995). Cervical approach for the repair of esophageal atresia. J Pediatr Surg.

[3] Fitoz S, Atasoy C, Yagmurlu A, Akyar S, Erden A, Dindar H (2000). Three-dimensional CT of congenital esophageal atresia and distal tracheoesophageal fistula in neonates: preliminary results. AJR Am J Roentgenol.

[4] Garge S, Rao KL, Bawa M (2013). The role of preoperative CT scan in patients with tracheoesophageal fistula: a review. J Pediatr Surg.

